# Supplementary material for: Deltamethrin-Evoked ER Stress Promotes Neuroinflammation in the Adult Mouse Hippocampus
Source: Cells. 2022 Jun 18;11(12):1961. doi: 10.3390/cells11121961 (PMC9222034; doi:10.3390/cells11121961)
Supplement: Supplementary file 1 [file cells-11-01961-s001.zip › cells-1741426-supplementary.pdf]

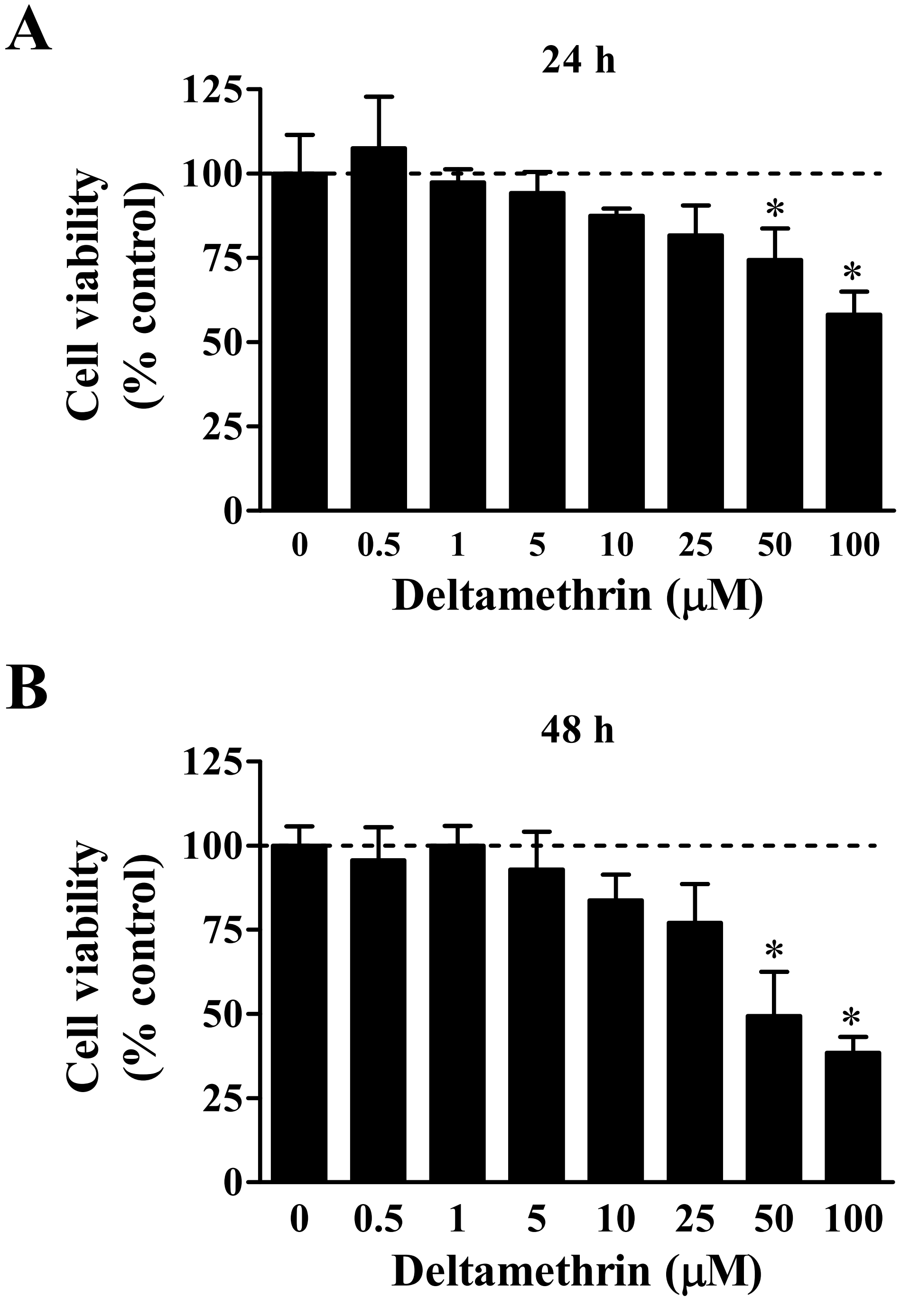

Supplementary Figure S1. Cytotoxic effect of deltamethrin on MMCs. Cells were treated with several concentrations of deltamethrin for 24 h (A) and 48 h (B). At the end of treatment, cell viability was evaluated using the AlamarBlue® cell viability reagent (Invitrogen, Grand Island, New York). Data represent mean ± SEM from 3 independent experiments, each of which was performed in triplicate. Asterisk denotes significant difference from control ($p<0.05$).
